# Clinical and cytopathological characteristics of HTLV‐1^+^ hodgkin lymphoma

**DOI:** 10.1002/cam4.3139

**Published:** 2020-06-28

**Authors:** Katsumi Kobata, Shoichi Kimura, Yasuhito Mihashi, Hiromi Iwasaki, Shuichi Nonaka, Shinji Matsumoto, Yasushi Takamatsu, Ilseung Choi, Shigeto Kawauchi, Kenji Ishitsuka, Morishige Takeshita

**Affiliations:** ^1^ Division of Diagnostic Pathology Faculty of Medicine Fukuoka University Hospital Fukuoka Japan; ^2^ Departments of Pathology Faculty of Medicine Fukuoka University Fukuoka Japan; ^3^ Departments of Otolaryngology Faculty of Medicine Fukuoka University Fukuoka Japan; ^4^ Departments of Hematology National Hospital Organization Kyushu Medical Center Fukuoka Japan; ^5^ Departments of Pathology National Hospital Organization Kyushu Medical Center Fukuoka Japan; ^6^ Division of Medical Oncology, Hematology and Infectious Diseases Faculty of Medicine Fukuoka University Fukuoka Japan; ^7^ Department of Hematology National Hospital Organization Kyushu Cancer Center Fukuoka Japan; ^8^ Department of Internal Medicine Faculty of Medicine Kagoshima University Kagoshima Japan

## Abstract

**Background:**

Human T‐lymphotropic virus‐1 (HTLV‐1)^+^ Hodgkin lymphoma (HL) is difficult to differentiate from adult T‐cell leukemia/lymphoma (ATLL) with HL‐like histology (HL‐like ATLL).

**Methods:**

Cytological and immunohistological features, HTLV‐1 proviral DNA integration, and rearrangements of the T‐cell receptor (TCR) *Cβ1* gene were examined in 11 HTLV‐1^+^ patients with HL‐like disease.

**Results:**

Six patients were classified as HTLV‐1^+^ HL and five as HL‐like ATLL in accordance with genetic findings of HTLV‐1 proviral DNA integration and rearrangements of the TCR *Cβ1* gene. Small ordinary looking lymphocytes with round nuclei were detected in the background of six patients with HTLV‐1^+^ HL, which were immunohistochemically negative for CD25 and CC chemokine receptor (CCR)4 and had a low MIB1 labeling index (mean: 28.3%). In the HL‐like ATLL specimens, small‐ and medium‐sized atypical lymphocytes with indented and irregular‐shaped nuclei were found, and were diffusely positive for CD25 and CCR4, with high MIB1 labeling (mean: 76%). Both groups had scattered CD30^+^ and CD15^+^ Hodgkin and Reed Sternberg (RS) giant cells, with or without CD20 expression and Epstein‐Barr virus infection. The 50% overall survival period was significantly longer for the HTLV‐1^+^ HL group (180 months) than for the HL‐like ATLL group (7.8 months; *P* = .004).

**Conclusions:**

HTLV‐1^+^ HL showed typical small lymphoid cells with a low MIB1 labeling index in a background of Hodgkin and RS cells, with some scattered CD25^+^ and CCR4^+^ lymphocytes. In HTLV‐1 endemic areas, distinguishing HTLV‐1^+^ HL from HL‐like ATLL is important because of their differing treatment strategies and prognoses.

## Graphical abstract

Human T‐lymphotropic virus‐1 (HTLV‐1)^+^ Hodgkin lymphoma (HL) is difficult to differentiate from adult T‐cell leukemia/lymphoma with HL‐like histology. Here, we compared cytohistology, clinicopathological features and prognoses between patients with genetically confirmed HTLV‐1^+^ HL and those with HL‐like ATLL.

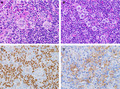

## 1 INTRODUCTION

Adult T‐cell leukemia/lymphoma (ATLL) mainly consists of CD4^+^ T‐cell neoplasms of the peripheral blood and lymph nodes, and is associated with long‐standing human T‐lymphotropic virus‐1 (HTLV‐1) infection.^1^ ATLL is characterized by a high likelihood of leukemic changes—acute and lymphoma ATLL subtypes have a progressive clinical course.^2^ Histologically, ATLL is classified into pleomorphic small‐, medium‐, and large‐sized lymphoma type, CD30^+^ anaplastic large cell (ALC), and rarely as Hodgkin lymphoma (HL)‐like.^1^

HL‐like ATLL is rare and displays scattered CD30^+^ and CD15^+^ Hodgkin and Reed Sternberg (RS) cells, often with Epstein‐Barr virus (EBV) infection against a background of small‐ and medium‐sized lymphocytes with atypical nuclear contours.^3^, ^4^, ^5^, ^6^ Both HL‐like ATLL and the more common pleomorphic‐type ATLL show integration of *HTLV‐1* proviral DNA and rearrangements of the T‐cell receptor (*TCR) Cβ1* gene in tumor tissues. The 5‐year survival of patients with HL‐like ATLL is 25.9%, which is greater than that of the other ATLL histological subtypes.^7^ However, HL patients are rarely found to be HTLV‐1 carriers, and differentiating between HL, HL‐like, and ALC type ATLL, as well as HTLV‐1^−^ peripheral T‐cell lymphoma (PTCL) with HL‐like features by histological findings alone is difficult.^8^, ^9^, ^10^, ^11^ The current study differentiated HTLV‐1^+^ HL from HL‐like ATLL by examining *HTLV‐1* proviral DNA integration and *TCR Cβ1* gene rearrangements in tumor specimens. The two groups were found to differ in clinicopathological features, cytological findings, and prognoses.

## 2 MATERIALS AND METHODS

### 2.1 Patient Selection and clinical findings

Clinicopathological records and samples of HTLV‐1 carriers who were diagnosed as ATLL (165 patients) and HL‐like lymphoproliferative disease (20 patients) between 1990 and 2019 were retrospectively retrieved from the Department of Pathology at Fukuoka University. This study focused on 11 HTLV‐1^+^ patients with cytohistological and genetic examinations in tumor tissues, in which Hodgkin and RS‐like cells were intermingled. Clinical subtypes of ATLL were classified according to the Shimoyama classification criteria.^2^ Clinical information was obtained by reviewing patient medical records. Histological classification was performed according to the 2017 WHO guidelines.^1^, ^3^, ^8^ Institutional ethical approval was obtained in compliance with the Declaration of Helsinki (institutional review board approval number: U19‐08‐014).

### 2.2 Cytology

Fine‐needle aspiration and tumor touch smear samples were obtained in all 11 HTLV‐1 carriers with HL‐like histology prior to initial treatment. Fine‐needle aspiration was performed using 10‐ml disposable syringes with 22‐ to 23‐gauge disposable needles. Stump specimens from surgically excised samples of lymph nodes and skin tumors were used in the study. Papanicolaou (Pap) staining was performed for methanol‐fixed specimens, and air‐dried specimens were stained by the May‐Giemsa method. Slides from selected specimens were reviewed by three cytologists. Nuclear size, nuclear contours, nuclear chromatin patterns (fine granular, coarse granular, stippled) and nucleoli of lymphoid cells, and giant nuclear neoplastic cells were cytologically analyzed, mainly using the Pap stain. Nuclear diameter was measured using scale bars in the Pap stained samples.^12^, ^13^ Cells with a nuclear diameter smaller than 5 µm were tentatively defined as small lymphocytes, irrespective of whether they were nonneoplastic (normal) or neoplastic. Lymphocytes with nuclei of 5‐8 µm and greater than 8 µm in diameter were defined as medium‐sized and large lymphocytes, respectively. Small lymphocytes with round hyperchromatic nuclei and indistinct nucleoli were considered normal small lymphocytes. Lymphoid cells with nuclei larger than 13 µm in diameter were defined as giant cells.

### 2.3 Histology, Immunohistology, and Detection of EBV‐encoded RNA

Fourteen excised lymph nodes and skin tumor specimens from the 11 examined patients were fixed in 10% formalin, embedded in paraffin tissue blocks and then stained with hematoxylin and eosin. For immunohistology, monoclonal and polyclonal antibodies were applied to formalin‐fixed tumor samples using a Leica Bond III automated stainer (Leica Biosystems) and Leica's proprietary antigen retrieval solution—the peroxidase reaction was developed using diaminobenzidine as the substrate.^14^ Immunostaining was performed using antibodies against CD3 (PS1, Leica Biosystems), CD4 (4B12, Leica Biosystems), CD8 (1A5, Leica Biosystems), CD25 (4C9, Leica Biosystems), CC chemokine receptor (CCR)4 (1G1, Bioscience), CD20 (L26, Nichirei), MIB1 (MIB1, DakoCytomation), CD30 (BerH2, DakoCytomation), CD15 (LeuM1, DakoCytomation), PAX5 (Thermo Scientific), PD‐L1 (E1L3N, Cell Signaling), and LMP1 (CS1‐4, DakoCytomation). Samples in which ≥ 30% of tumor cells were labelled with a specific antibody were considered positive. Identification of Hodgkin and RS cells was determined by CD30 and CD15 expressions and negative CD3, as well as by histology. The presence of EBV infection was determined by the in situ hybridization of EBV‐encoded RNA (EBER)(+) nuclear signals. Deparaffinized tissue sections were digested with proteinase K and hybridized in 50% formamide containing fluorescein isothiocyanate‐labeled EBER oligonucleotides (BOND EBER probe, Leica Biosystems).

### 2.4 Southern Blot Analysis (SBA) of immuno‐associated genes

High molecular weight DNA was extracted from fresh tumor specimens and subjected to Southern blot analysis as described previously.^15^ Briefly, 10 µg of DNA digested with *Eco*RI and *Pst*I for the *HTLV‐1* proviral DNA, and *Eco*RI, *Hin*dIII and *Bam*HI for the *TCR Cβ1* gene, were electrophoresed on 0.7% agarose gels, transferred to a nitrocellulose filter, and hybridized to ^32^P‐labeled DNA probes. Examined probes were labeled using a DNA random primer kit (Amersham). DNA rearrangement was scored on the basis of loss of the germ line band (deletion) or on the appearance of newly rearranged bands.

### 2.5 Statistical analysis

All pairwise comparisons of categorical variables of clinicopathological characteristics among the two groups were performed using the chi‐square test or Fisher's extract test. Patient outcomes were determined by calculating cumulative survival from time of diagnosis to the date of last follow‐up or death. For survival analyses, our study was limited to patients for whom follow‐up medical information and outcomes were available. Overall survival (OS) curves were generated using the Kaplan‐Meier method and compared using the Cox proportional hazard model. Statistical analyses were performed using JMP 10 software (SAS Institute). *P* < .05 was considered statistically significant.

## 3 RESULTS

### 3.1 Genetic studies

**FIGURE 1:**
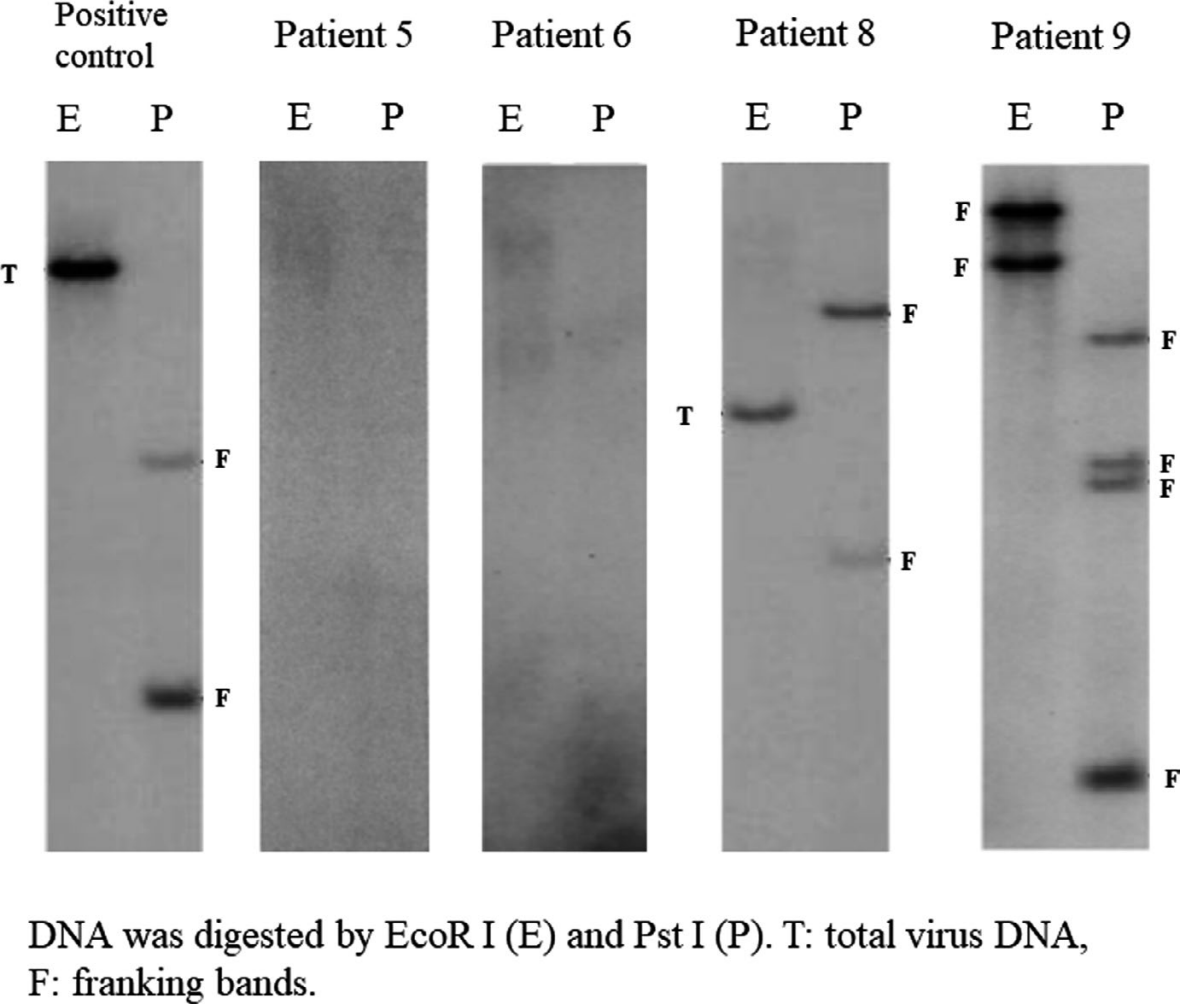
Integration of *HTLV‐1* proviral DNA. Two patients (5 and 6) with HTLV‐1^+^ Hodgkin lymphoma show no integration of *HTLV‐1* proviral DNA; two patients (8 and 9) with Hodgkin lymphoma‐like adult T‐cell leukemia/lymphoma have franking integration bands (F) of *HTLV‐1* proviral DNA

**FIGURE 2:**
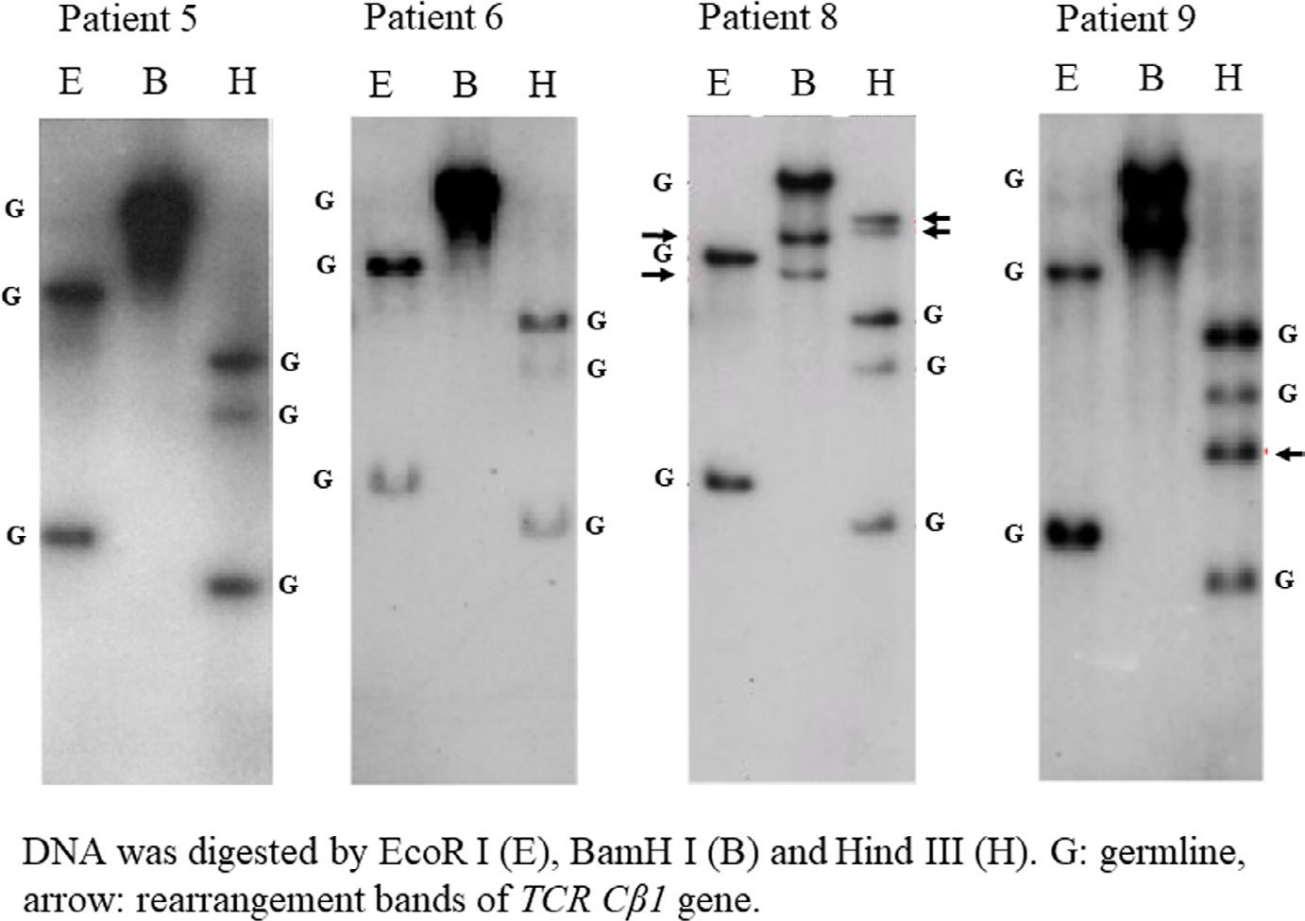
Examination of *TCR Cβ1* gene rearrangements. Two patients (5 and 6) with HTLV‐1^+^ Hodgkin lymphoma show germline (G) configurations of the *TCR Cβ1* gene. Two patients (8 and 9) with Hodgkin lymphoma‐like adult T‐cell leukemia/lymphoma show rearrangement bands (arrows)

Frozen samples from six patients, including samples of recurrence in two patients (Patients 2 and 3), showed no integration of *HTLV‐1* proviral DNA and germline configurations of *TCR Cβ1,* and were classified as HTLV‐1^+^ HL. No genetic studies were performed at conversion point to acute type ATLL in Patient 1. The remaining five patients had integrated *HTLV‐1* proviral DNA and *TCR Cβ1* gene rearrangements, and were classified as HL‐like ATLL. Representative results are shown in Figures 1 and 2.

### 3.2 Clinical findings

**FIGURE 3:**
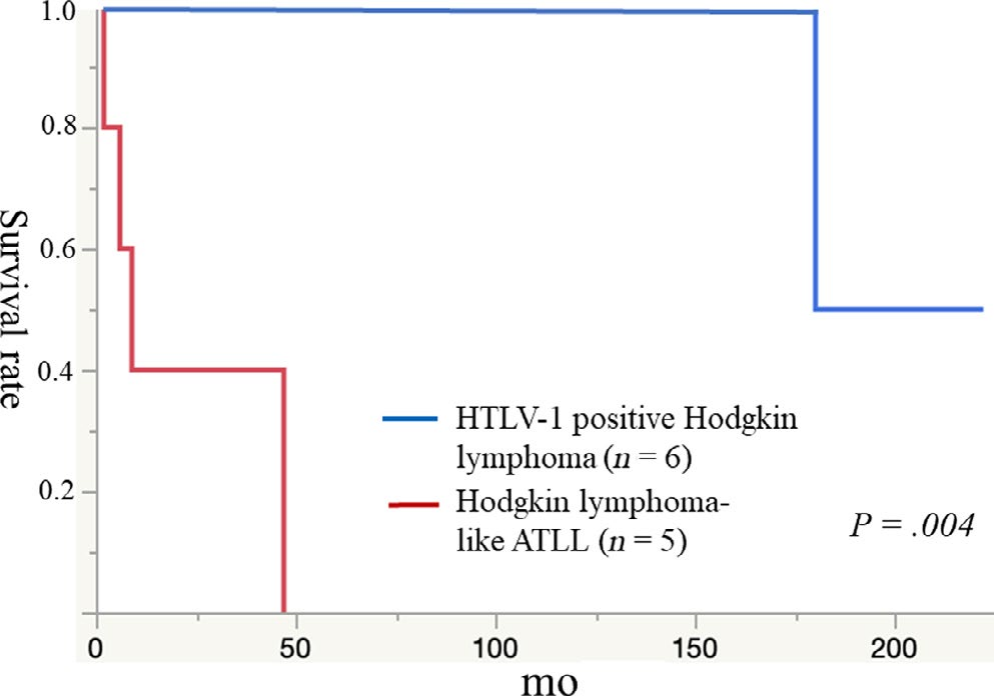
Patients with HTLV‐1^+^ Hodgkin lymphoma (n = 6) have significantly better overall survival than patients with Hodgkin lymphoma‐like adult T‐cell leukemia/lymphoma (n = 5; *P* = .004)

Clinical findings, treatments, and prognoses are shown in Table 1. In the HTLV‐1^+^ HL group (n = 6), median age at diagnosis was 57.3 years—two patients had HL nodular sclerosis subtype and four had mixed cellularity. High LDH (>400 U/L) was found in one of six patients with HTLV‐1^+^ HL and three of five patients with HL‐like ATLL. Elevated soluble interleukin 2 receptor (sIL2R, >2000 U/mL) was not found in four patients with HTLV‐1^+^ HL, but was detected in three patients with HL‐like ATLL (*P* = .029). Five of six HTLV‐1^+^ HL patients were classified as Ann Arbor clinical stage I or II disease by systemic imaging studies. The remaining patient (Patient 3) had stage IV disease due to bone marrow invasion with some scattered giant cells and severe collagenous fibrosis. Five HTLV‐1^+^ HL patients received adriamycin, bleomycin, vinblastine, and dacarbazine (ABVD) and/or local radiation at initial diagnosis. Patient 1 converted to acute‐type ATLL with overt leukemic changes, skin and colonic tumor invasion after 44 months. He received mogamulizumab (anti‐CCR4) and sobuzoxane, and is disease‐free at 60 months. Two other patients had recurrences of HL. Patient 2 was histologically misdiagnosed with ALC‐type ATLL at her second recurrence, for which she received a therarubicin (THP), vincristine, endoxan, prednisolone, and adriamycin (VEPA) regimen. After 10 years, she relapsed and received VEPA, and is disease‐free at 222 months. Patient 3 underwent allogenic bone marrow transplantation for recurrence at 84 months, and THP, cyclophosphamide, vincristine, and prednisolone (THP‐COP) for recurrence at 134 months. She achieved complete remission, but finally died of disease at 180 months. In the HL‐like ATLL group (n = 5), the median age was 64.2 years—four patients had lymphoma‐type ATLL and one had acute‐type ATLL. Of the 11 patients, only one HL‐like ATLL patient (Patient 10) showed hypercalcemia (10.8 mg/dL). All five patients with HL‐like ATLL had stage III or IV disease, and received either a cyclophosphamide, doxorubicin, vincristine, and prednisolone (CHOP) regimen or VEPA therapy. Patient 9 was additionally treated with mogamulizumab (anti‐CCR4) and is alive at 26 months. The remaining four patients died of disease at 2‐47 months. The 50% OS in the HTLV‐1^+^ HL group (180 months) was significantly longer than that in the HL‐like ATLL group (7.8 months; *P* = .004; Figure 3).

**TABLE 1:** Clinical characteristics of 11 patients with HTLV‐1 positive Hodgkin lymphoma and adult T‐cell leukemia/lymphoma

Pt.	Age (yr)	Sex	WBC/µL	Atypical lymphocytes (%)	LDH (U/L)	sIL2‐R (U/mL)	Clinical stage	Histology	*HTLV‐1* proviral DNA	*TCR Cβ1* gene	Therapies	Outcome
1	66	M	7200	1	164	1012	IIA	HL, NS	—	GL	ABVD	60, alive
	69^a^		26 000	53	348	23 707	IV	ATLL	nt	nt	Mogamulizumab + Sobuzoxane	
2	28	F	5300	2	321	nt	IA	HL, NS	—	GL	Radiation (THP‐VEPA^b^)	222, alive
	38^a^		3980	0	445	1110	IIA	HL, NS	—	GL	VEPA	
3	44	F	3600	0	809	nt	IV	HL, MC	—	GL	ABVD (Allo BMT^c^)	180, dead
	55^a^		4300	0	336	5250	IV	HL, MC	—	GL	THP‐COP	
4	68	F	5900	0.5	216	587	IA	HL, MC	—	GL	ABVD + Radiation	80, alive
5	72	M	5700	0	189	781	IA	HL, MC	—	GL	ABVD	64, alive
6	62	M	5900	0.5	240	1954	IIA	HL, MC	—	GL	ABVD	12, alive
7	54	M	4100	1	746	nt	IIIA	ATLL, HL‐like	I	R	VEPA	47, dead
8	66	F	16 200	0	307	10800	IV	ATLL, HL‐like	I	R	CHOP	9, dead
9	65	F	5900	0.5	254	2232	IIIA	ATLL, HL‐like	I	R	CHOP + Mogamulizumab	26, alive
10	67	M	6200	0	1861	9204	IV	ATLL, HL‐like	I	R	CHOP	2, dead
11	69	M	20 100	70	1413	nt	IV	ATLL, HL‐like	I	R	VEPA	6, dead

Abbreviations: ABVD, adriamycin, bleomycin, vinblastine, dacarbazine; CHOP, cyclophosphamide, doxorubicin, vincristine, prednisone; GL, germ line; I, integration; nt, not tested; Pt., patient; R, rearranged; THP, therarubichin; VEPA, vincristine, cyclophosphamide (endoxan), prednisolone,Adriamycin.

^a^ Data in recurrence.

^b^ THP‐VEPA treatment was performed at second relapse

^c^ Allo BMT: allogenic bone marrow transplantation was performed in recurrence at 84 mo after initial presentation.

### 3.3 Cytological and histological findings

**FIGURE 4:**
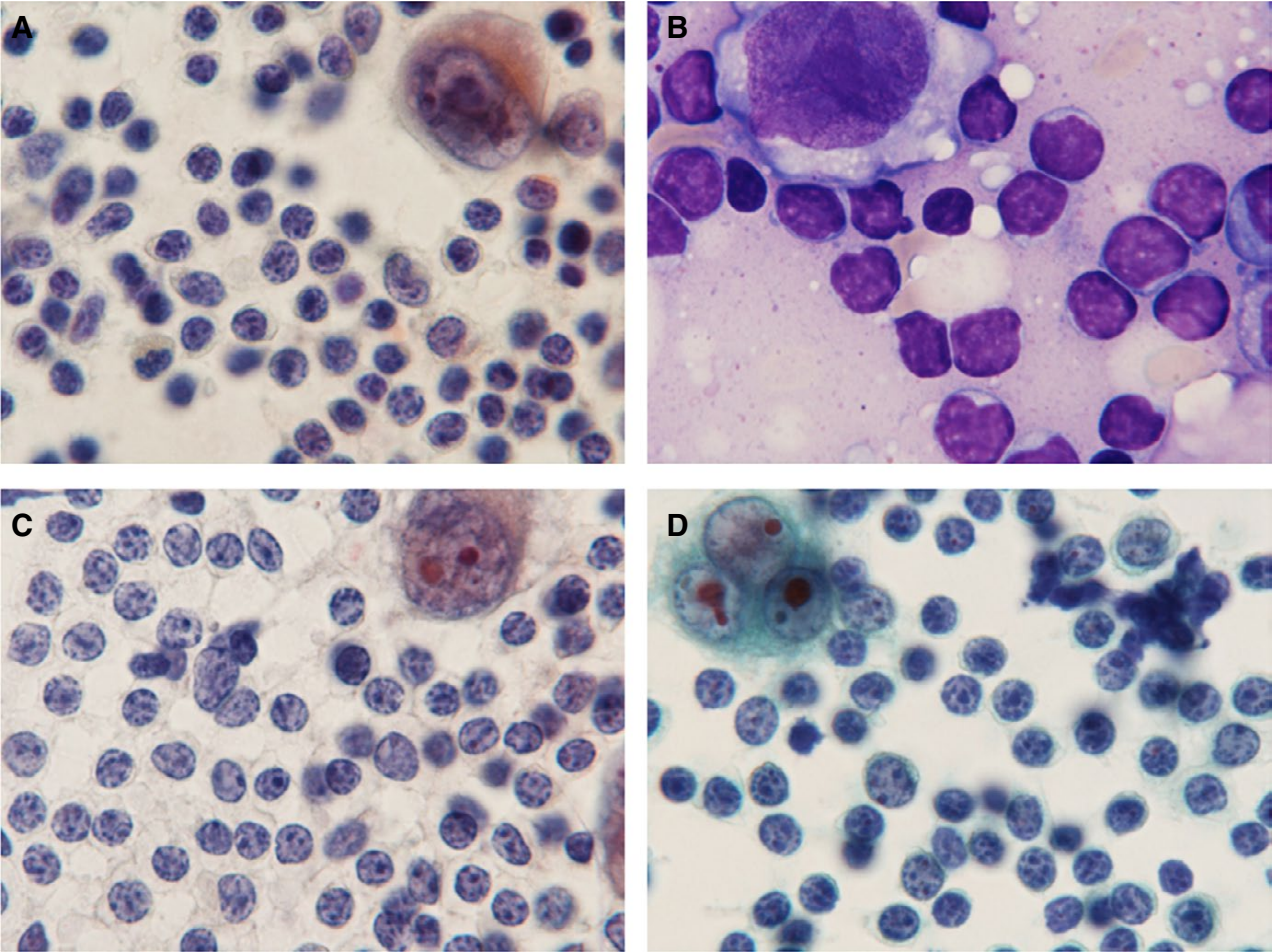
HTLV‐1^+^ Hodgkin lymphoma cytology. A, B: Many small lymphocytes with round nuclei and coarse chromatin, and Hodgkin or Reed Sternberg cells with large nucleoli were found. A, C, D: Pap stain, B: Giemsa stain × 1000. A, B: Patient 1, C: Patient 3, D: Patient 6

**FIGURE 5:**
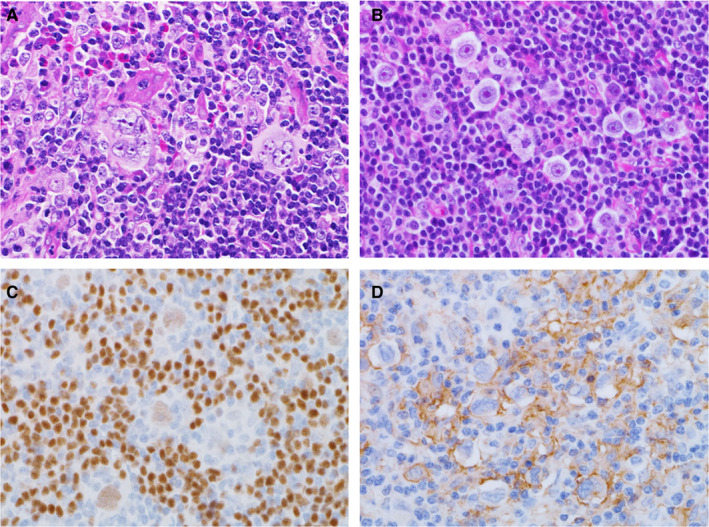
A, B: HTLV‐1^+^ Hodgkin lymphoma histology. Scattered Hodgkin and Reed Sternberg cells, many round nuclear small lymphocytes and some eosinophils in the background. H.E stain. Immunohistological stains show scattered PAX5^+^ (C) and PD‐L1^+^ (D) giant nuclear cells. Many PAX5^+^ small B cells are distributed throughout the sample, ×600. A, D: Patient 1, B: Patient 6, C, Patient 3

**FIGURE 6:**
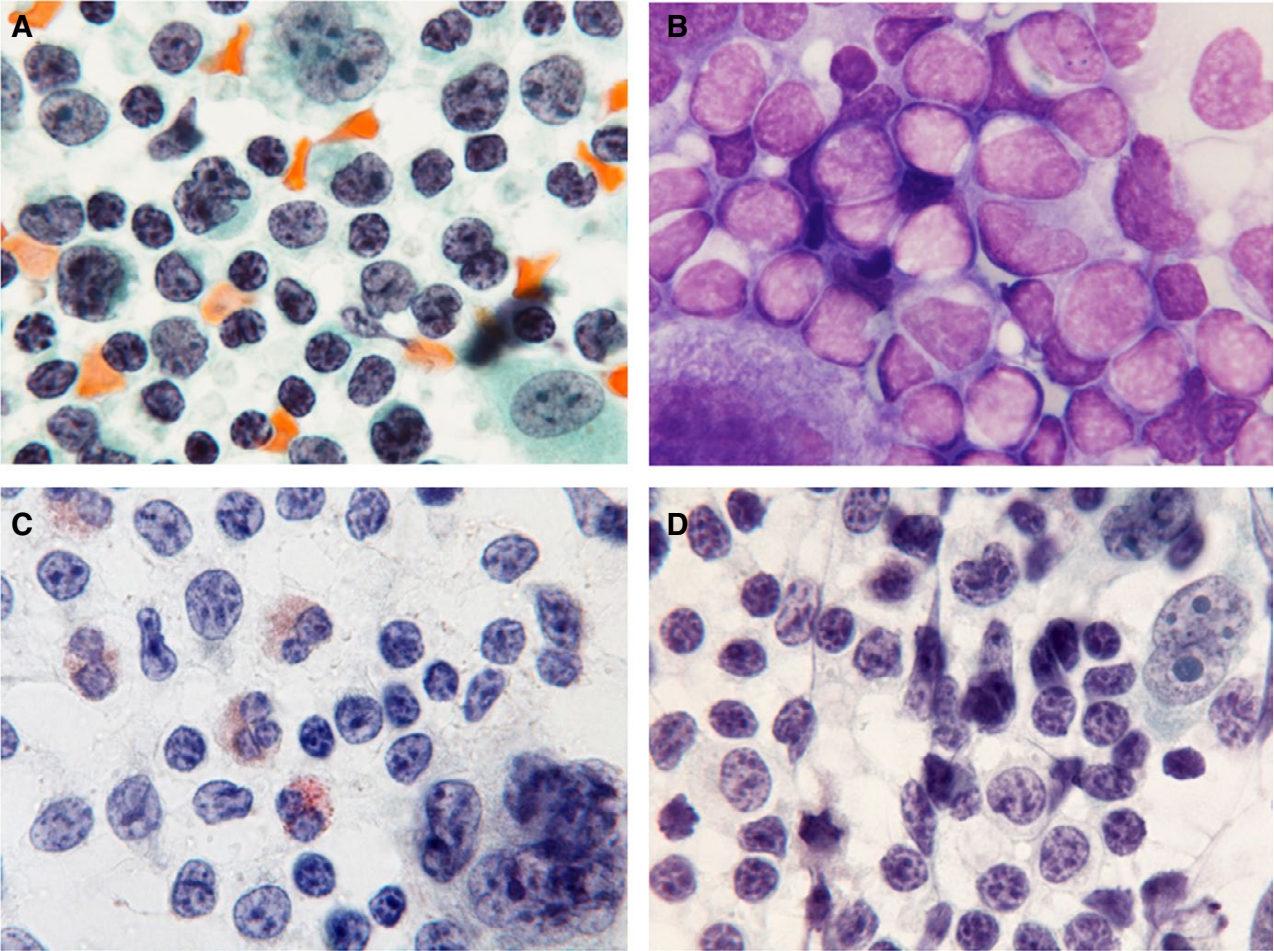
A‐D, Hodgkin lymphoma‐like adult T‐cell leukemia/lymphoma cytology. Lymphocytes with irregular nuclear contours, coarse and stippled chromatin were detected, including some with indented nuclei. Hodgkin and Reed Sternberg cells with large distinct nucleoli were seen. A, C, D, Pap stain, B, Giemsa stain × 1000. A, B, Patient 7, C: Patient 8, D: Patient 9

**FIGURE 7:**
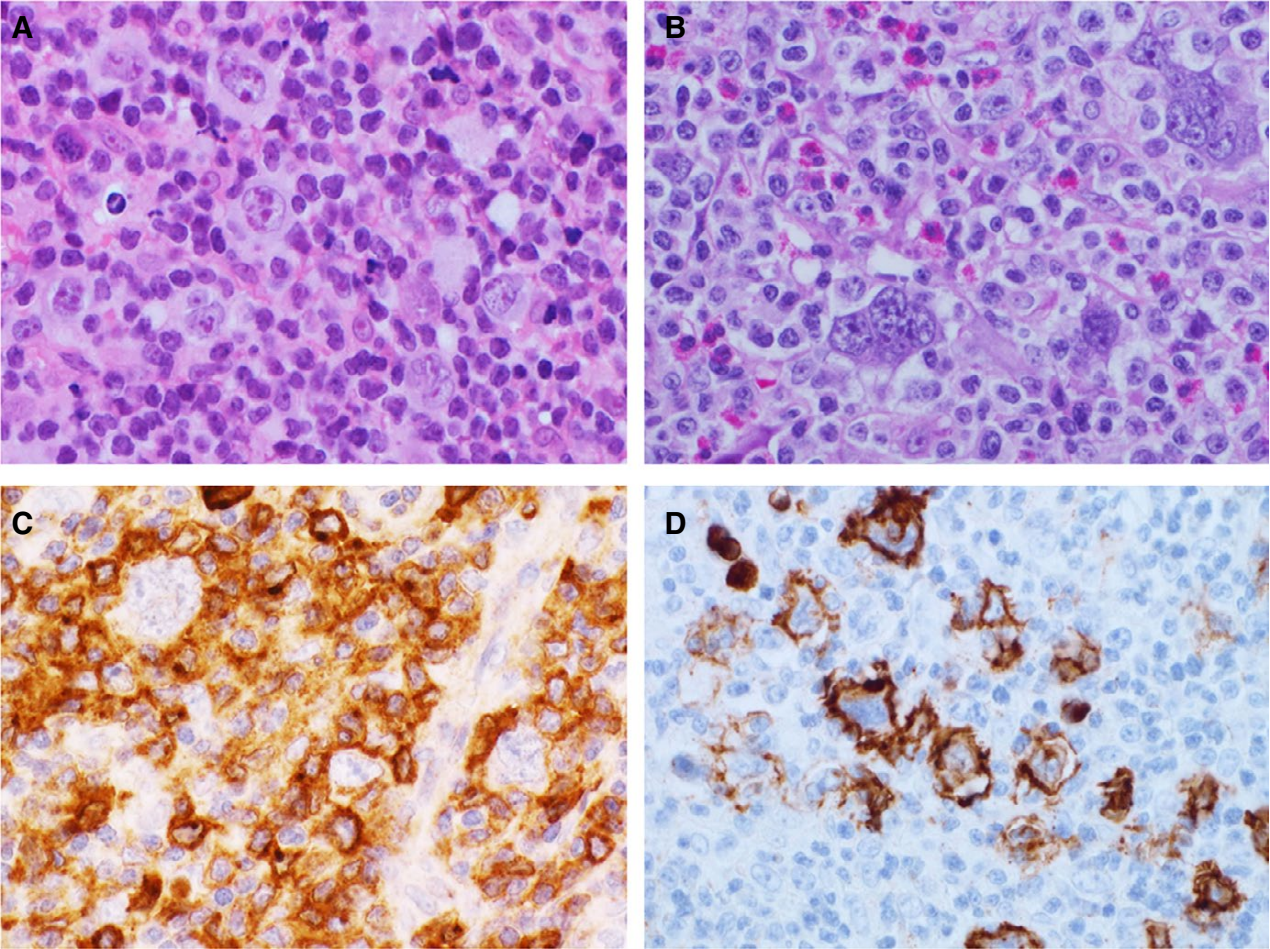
A, B, Hodgkin lymphoma‐like adult T‐cell leukemia/lymphoma with some scattered Hodgkin and Reed Sternberg cells by histology. Small‐ and medium‐sized lymphoid cells with irregular nuclear contour were also seen. HE stain, ×600. C, Many CCR4^+^ lymphoid cells are present in the background of giant cells. D, Some CD20^+^ giant cells are scattered, ×600. A: Patient 7, B, C: Patient 8, D: Patient 10

In the HTLV‐1^+^ HL patients, many small lymphoid cells with round nuclei and coarse chromatin were detected in a background of Hodgkin and RS cells with distinct large nucleoli (Pap and Giemsa stains; Figure 4A‐D). Nuclear indentation and pleomorphism, and irregular nuclear contours were indefinite among Pap‐stained infiltrating small lymphoid cells. Histologically, samples from HTLV‐1^+^ HL patients showed diffuse infiltrates of small lymphocytes with round nuclei and coarse chromatin against a background of scattered Hodgkin and RS cells with large distinct nucleoli (Figure 5A,B). Eosinophilic infiltrates were detected in five HTLV‐1^+^ HL patients (83.3%), plasma cells were detected in four (66.7%), and histiocytes were present in all six (100%). Severe and patchy fibrosis was observed in three patients (50%) and preserved lymph follicles were present in five (83.3%). In samples from the HL‐like ATLL group, many small‐ and medium‐sized lymphoid cells had mildly irregular shaped nuclei and coarse chromatin, and some showed nuclear indentation (Pap and Giemsa stains; Figure 6A‐D). Some large atypical lymphoid cells with coarse and stippled chromatin and small nucleoli, were intermingled. Scattered Hodgkin and RS cells had distinct large nucleoli that were similar to those of giant cells found in the HTLV‐1^+^ HL group. Histologically, HL‐like ATLL patients had many small‐ and medium‐sized lymphoid cells with mildly irregular or indented nuclei in a background of Hodgkin and RS cells (Figure 7A,B). Eosinophilic and plasma cell infiltrates were detected in three HL‐like ATLL patients (60%) and histiocytes were detected in all five (100%). Patchy fibrosis was detected in three patients (60%) and preserved lymph follicles were observed in two (40%). Lower numbers of eosinophils, plasma cells, and lymph follicles were observed in HL‐like ATLL compared with HTLV‐1^+^ HL.

### 3.4 Immunohistological findings

Immunohistological characteristics of background lymphoid cells and specific giant cells in the two groups are shown in Table 2. Infiltrating lymphocytes in both groups were mainly CD3^+^ and CD4^+^. In HTLV‐1^+^ HL patients, many CD8^+^ T cells were intermingled (n = 4, 66.7%) or scattered (n = 2, Patients 2 and 3), and many CD20^+^ lymphocytes were scattered and clustered in all six patients (100%). CD25^+^ and CCR4^+^ lymphoid cells were not found or were scattered in HTLV‐1^+^ HL samples. Background lymphoid cells in HL‐like ATLL were diffusely positive for CD25 and CCR4 (Figure 7C). In one patient with HL‐like ATLL (20%), many CD20^+^ B cells were clustered in the involved lesions. The mean MIB1 labeling index of background lymphoid cells was significantly lower in the HTLV‐1^+^ HL group (28.3%) than in the HL‐like ATLL group (78%; *P* < .043). In HTLV‐1^+^ HL patients, scattered and partially clustered Hodgkin and RS cells were CD15^+^, CD30^+^, and CD3^−^ in all 11 patients, weakly positive for PAX5 and/or CD20 in three, and PD‐L1^+^ in four (Figure 5C,D). CD20^+^, PAX5^+^, and PD‐L1^+^ giant cells were detected in two, three, and three HL‐like ATLL patients, respectively (Figure 7D). LMP1^+^, EBERs^+^, and EBNA2^−^ giant cells were found in three HTLV‐1^+^ HL patients and four HL‐like ATLL patients.

**TABLE 2:** Histological and immunohistological findings in 11 patients with HTLV‐1 positive Hodgkin lymphoma and adult T‐cell leukemia/lymphoma

Pt	Lymphocytes in background							Hodgkin and Reed Sternberg cells								
	CD3	CD4	CD8	CD20	CD25	CCR4	MIB‐1	CD30	CD15	CD20	PAX‐5	CD25	PD‐L1	CD3	LMP1	EBERs
1	＋	＋	＋	＋	－	－	+(30%)	＋	＋	－	＋	＋	+(50%)	－	－	－
1^a^	＋	＋	－	－	＋	＋	+(80%)	ne	ne	ne	ne	ne	ne	Ne	ne	ne
2	＋	＋	－^b^	－	－	－	+(30%)	＋	＋	－	－	－	+(50%)	－	－	－
2^a^	＋	＋	－^b^	－	－	－	‐(20%)	＋	＋	－	－	＋	+(50%)	－	－	－
3	＋	＋	－^b^	－	－	－	‐(20%)	＋	＋	－	＋	－	+(50%)	－	＋	＋
3^a^	＋	＋	＋	＋	－	－	+(30%)	＋	＋	＋	＋	－	+(40%)	－	＋	＋
4	＋	＋	＋	＋	－	－	+(30%)	＋	＋	－	－	－	－	－	－	－
5	＋	＋	＋	＋	－	－	+(30%)	＋	＋	－	－	－	－	－	＋	＋
6	＋	＋	＋	＋	－	－	+(30%)	＋	＋	－	＋	－	+(30%)	－	＋	＋
7	＋	＋	＋	－	＋	＋	+(90%)	＋	＋	－	－	－	+(90%)	－	－	－
8	＋	＋	－	－	＋	＋	+(70%)	＋	＋	＋	＋	－	－	－	＋	＋
9	＋	＋	－	－^c^	＋	＋	+(70%)	＋	＋	－	＋	－	+(50%)	－	＋	＋
10	＋	＋	－	－	＋	＋	+(80%)	＋	＋	＋	＋	－	－	－	＋	＋
11	＋	＋	－	－	＋	＋	+(80%)	＋	＋	－	－	－	+(50%)	－	＋	＋

Abbreviations: ne: not evaluated; Pt., patient.

^a^ Data in recurrence.

^b^ CD8‐positive cells are scattered.

^c^ Many CD20‐positive reactive B cells are scattered and clustered.

## 4 DISCUSSION

Genetic studies of integrated *HTLV‐1* proviral DNA and rearranged *TCR Cβ1* gene are indispensable to diagnosing ATLL. In a previously reported cohort of nine HTLV‐1^+^ HL patients,^8^, ^16^, ^17^, ^18^ only one showed no integration of *HTLV‐1* proviral DNA, had a germline configuration of the *TCR Cβ1* gene, and was still well months after six courses of CHOP.^8^ Another three patients showed integrated *HTLV‐1* proviral DNA, but their *TCR Cβ* gene was not examined,^16^, ^18^ of these, two died of disease with conversion to ATLL after 10 months and 3 years, and the remaining patient was alive at 42 months. The other five patients received no genetic examinations,^17^, ^18^ among them, four were alive at 10‐60 months and one died of disease after 1 month. These findings suggest that HTLV‐1^+^ HL‐like lesions with integrated *HTLV‐1* proviral DNA indicate ATLL,^15^ on the basis of their progressive clinical courses. Because HTLV‐1 carriers occasionally suffer from follicular and diffuse large B‐cell lymphomas,^19^, ^20^ six HTLV‐1^+^ HL patients with no integration of *HTLV‐1* proviral DNA, and germline configuration of the *TCR Cβ1* gene were also evaluated. Constant examinations of both types of genetic study are necessary to confirm ATLL in HTLV‐1^+^ HL‐like lesions and recurrent tumors after treatment because of the possibility of conversion to ATLL.

Ohshima et al reported the respective 2‐ and 5‐year OS rates of 18 patients with HL‐like ATLL were 62.1% and 25.9% suggesting HL‐like ATLL was a prodromal stage of ATLL.^7^ The current study found that the 50% OS of the HTLV‐1^+^ HL group was 180 months. One HTLV‐1^+^ HL patient showed overt leukemic conversion with skin and colonic tumor invasion at 44 months, but then passed 60 months in remission. In this study, HTLV‐1^+^ HL and HL‐like ATLL were considered to be completely different diseases. Furthermore, it was difficult to confirm whether HL‐like ATLL is a prodromal condition because all five patients had advanced‐stage disease and a progressive clinical course. A further cumulative study of HTLV‐1^+^ HL‐like lesions is necessary to confirm the detailed clinicopathological and genetic findings.

When Hodgkin and RS cells and granulomatous reactions are seen in ATLL and PTCL tissues, it is necessary to check for nuclear atypia in background lymphoid cells, populations of CD4^+^/CD8^+^ T cells, follicular helper T‐cell markers, and rearranged *TCR Cβ1* genes to differentiate HL.^10^, ^11^ Cytologically, background lymphocytes from HTLV‐1^+^ HL patients showed small round nuclei and coarse chromatin, which differ from small‐ and medium‐sized lymphoid cells with indented and lobulated nuclei, coarse and stippled chromatin in HL‐like ATLL. Furthermore, HTLV‐1^+^ HL patients mainly showed admixed features of CD4^+^ and CD8^+^ T cells without CD25 and CCR4 expressions, which are typical cell markers for ATLL, and a low mean MIB1 labeling index (28.3%). However, Hodgkin and RS cells found in both groups showed similar cytological and immunohistological findings to those of HTLV‐1^−^ HL. The current study clearly confirmed the cytopathological characteristics of background lymphocytes and specific giant cells in HTLV‐1^+^ HL.

Hodgkin and RS cells in HL‐like ATLL show frequent EBV infection by in situ hybridization, and single‐cell PCR of giant cells and detection of recombinase‐activating gene frequently showed immature B‐cell characteristics related to rearrangements of *IGH* and *JH* genes in B cells.^3^ Hodgkin and RS cells found in HTLV‐1^+^ HL and HL‐like ATLL did not express T‐cell markers or CCR4—giant cells in three patients in each group showed weakly positive reactions to CD20 and/or PAX5. Hodgkin and RS cells found in HTLV‐1^+^ HL‐like lesions were suggested to have B‐cell characteristics similar to those in HTLV‐1^−^ HL.

Hodgkin and RS cells, especially with EBV infection in HL, frequently express PD‐L1. Therefore, patients with therapy‐resistant HL have received anti‐PD1 therapy as well as brentuximab vedotin (anti‐CD30) in combination with chemo‐ and radiotherapies.^21^, ^22^ Because Hodgkin and RS cells in four patients with HTLV‐1^+^ HL and three with HL‐like ATLL expressed PD‐L1, local and systemic immune suppression against the specific giant cells might occur in HTLV‐1^+^ HL‐like lesions. Although six HTLV‐1^+^ HL patients showed complete remission after ABVD and/or local radiotherapy, four were older than 60 years and three showed conversion to ATLL or relapses of therapy‐resistant HL. New therapies, including immune checkpoint inhibitors and anti‐CD30, may help therapy‐resistant HTLV‐1^+^ HL patients.

The main limitation of this study was the small number of patients (n = 11) available to support our cytological and clinicopathological findings. Additional cases are necessary to confirm the relationship among the cytological, clinicopathological features, and prognoses between the two examined groups.

## 5 CONCLUSIONS

Patients with HTLV‐1^+^ HL are usually classified as early clinical stage I or II following an indolent clinical course. In HTLV‐1‐endemic areas, checking serum HTLV‐1 antibodies is important, even in young HL patients. *HTLV‐1* proviral DNA integration and *TCR Cβ1* gene rearrangement are fundamental to diagnosing ATLL. Detailed cytological studies of background lymphocytes and their markers (including CD25, CCR4 and MIB1) help distinguish between the two different disorders. Larger studies of HTLV‐1^+^ HL‐like lesions are necessary to verify their specific clinical features and prognosis.

## CONFLICTS OF INTEREST

The authors declare that they have no significant relationships with, or financial interest in, any commercial activities pertaining to this article.

## AUTHORS’ CONTRIBUTIONS

K. K. and M. T. designed and performed the research. K. K., S. N., and S. M. analyzed cytological data. S. K., Y. M., S. K., and M. T. collected and summarized pathological data, and conducted the statistical analysis. H. I., Y. T., and K. I. conducted treatment and collected clinical data. K. K., S. K., and M. T. wrote the manuscript.
